# The influence of lifestyle and gender on sickness absence in Brazilian workers

**DOI:** 10.1186/1471-2458-14-317

**Published:** 2014-04-06

**Authors:** Fabiana Maluf Rabacow, Renata Bertazzi Levy, Paulo Rossi Menezes, Olinda do Carmo Luiz, Ana Maria Malik, Alex Burdorf

**Affiliations:** 1 Department of Preventive Medicine, University of São Paulo, School of Medicine, FMUSP, Avenida Dr Arnaldo, 455–2° andar, 01246-903 São Paulo, SP, Brazil; 2 Department of Public Health, Erasmus MC, University Medical Center Rotterdam, P.O. Box 2040, 3000, CA, Rotterdam, The Netherlands

**Keywords:** Sick leave, Gender, Health behavior

## Abstract

**Background:**

Despite an increasing body of knowledge concerning gender and lifestyle factors as determinants of sickness absence in well-developed countries, the relationship between these variables has not been elucidated in emerging economic power countries, where the burden of non-communicable diseases is particularly high. This study aimed to analyze the relationships among lifestyle-related factors and sick leave and to examine whether gender differences in sickness absence can be explained by differences in socio-demographic, work and lifestyle-related factors among Brazilian workers.

**Methods:**

In this longitudinal study with a one year follow-up among 2.150 employees of a Brazilian airline company, sick leave was the primary outcome of interest. Independent variables collected by interview at enrolment in the study were gender, age, educational level, type of work, stress, and lifestyle-related factors (body mass index, physical activity and smoking). In addition, the risk for coronary heart disease was determined based on measurement of blood pressure, total cholesterol and glucose levels. The total number of days on sick leave during 12 months follow-up was available from the company register. Logistic regression analysis was used to determine the influence of socio-demographic, type of work and lifestyle-related factors on sick leave.

**Results:**

Younger employees, those with lower educational level, those who worked as air crew members and those with higher levels of stress were more likely to have sick leave. Body mass index and level of physical activity were not associated with sick leave. After adjustment by socio-demographic variables, increased odds for 10 or more days of sick leave were found in smokers (OR = 1.51, CI = 1.05-2.17), and ex-smokers (OR = 1.45, CI = 1.01-2.10). Women were more likely to have 10 or more days of sick leave. Gender differences were reduced mainly when adjusted for type of work (15%) and educational level (7%).

**Conclusions:**

The higher occurrence of sick leave among women than among men was partly explained by type of work and educational level. Our results suggest that type of work, a stressful life, and smoking are important targets for health promotion in this study population.

## Background

Non-communicable diseases are the major cause of disability and premature mortality worldwide. The burden of non-communicable diseases is particularly high for emerging economic powers such as Brazil, Russia, India, and China, which together with South Africa are known as BRICS. Together, these countries currently lose more than 20 million productive life years annually to chronic diseases [1]. These diseases are due in large part to modifiable risk factors, such as smoking, physical inactivity, and poor dietary habits, beyond intermediate risk factors such as obesity, high blood pressure, cholesterol and glucose concentrations [2]. In Brazil, despite the successful implementation of health policies that led to a decrease in smoking (from 22.4% in 2003 to 14.8% in 2011) [3,4] and, consequently, declines of approximately 20% in cardiovascular and chronic respiratory diseases in recent years, the prevalence of diabetes and hypertension is rising in parallel with that of obesity. Between 2006 and 2011, the prevalence of obesity increased from 11.4% to 15.8% [4]. These increases are associated with unfavorable changes in diet and physical activity [5]. In the corporate setting, these risk factors, added to the rapid changes in working with more technology and increasing pressure on performance, may have consequences for workers’ health and productivity.

Several studies have investigated the relationship between absenteeism and modifiable risk factors, such as physical inactivity [6], obesity [7], inadequate nutrition [8], and smoking [9]. Some studies have reported increased absenteeism because of obesity [10] and lack of physical activity [11], whereas in other studies, obesity [7] and lack of physical activity [12] were not associated with absenteeism. It is unclear whether these contradictory findings are attributable to other differences, most notably gender, type of work, working conditions, and work-home interference.

In several European countries, women have higher rates of sickness absence than men [13-15]. Laaksonen et al. [16] found that occupation accounted for half of the female sick leave episodes lasting more than 60 days and approximately one third of sick leave less than 60 days. This study suggests that the type of work is an important mediating factor for gender differences in sick leave.

Despite an increasing body of knowledge concerning the relationship between lifestyle-related factors and absenteeism in well-developed countries, the current longitudinal study is one of the first studies in BRICS countries to investigate gender and lifestyle factors as determinants of sickness absence. The aims of this study were to analyze the relationship among lifestyle-related factors and sick leave and to examine whether gender differences in sickness absence can be explained by differences in type of work, educational level, and lifestyle factors.

## Methods

### Study design and participants

The company under study is a multinational airline company with over 28.000 employees in Brazil in 2013. Between May and November 2010, 3.147 employees of that company voluntarily participated in a survey conducted by a health insurance organization responsible for the health services for the company’s employees. Employees who were present and available on the dates of the assessment were evaluated. This assessment was conducted in the workplace and was structured as an interview conducted by a team of nurses. The questionnaire involved questions related to health and lifestyle. Weight and height were self-reported. In addition to the questionnaire, blood pressure, total cholesterol, and glucose levels were measured. Among the evaluated subjects, a linkage was made with the one-year sickness absence register from the company through the personal identification number of each employee. This procedure excluded 933 outsourced employees with insufficient information due to extended periods working outside the company, 13 pregnant women and 51 underweight (BMI < 18.5 Kgm^2^) workers. The study population consisted of 2.150 subjects, representing 16.2% of the company’s employees in São Paulo, Brazil. This project was submitted to the Ethics Committee in Research of the Medical School of Universidade de São Paulo, Brazil, and it was approved under research protocol number 083/12. The written consent of the participants was not obtained because this study used secondary data already collected. However, the company responsible for the data signed a written consent that information could be used for a research project. In the information that accompanied the questionnaire to participants, it was emphasized that privacy would be guaranteed and that all data would be treated confidentially and stored in secured computer systems. In accordance with Brazilian regulations, for the purpose of this study all identifiable information was completely removed before the dataset with anonymous data was provided to the first author.

### Sick leave

Information about sick leave was obtained from the records of the airline company for 12 months after enrolment in the study. Workers had to notify the company of sickness absence, but a specific diagnosis was only available for longer periods of absence, typically above two weeks and, hence, not considered for this study. Before we summed the number of sick leave days per worker for twelve months, it was possible to identify the pregnancy-related sick leaves and exclude them. All sickness absence episodes during the follow-up period were summed per worker and classified in three categories: no sick leave, 1–9 days of sick leave, and 10 days or more of sick leave.

### Independent variables

We considered as lifestyle-related factors body mass index (BMI), physical activity and smoking. Body mass index was calculated by dividing self-reported body weight (in kilograms) by self-reported height squared (in meters) and it was categorized as “normal weight” individuals with BMI between 18.5 to 24.9 kg/m^2^, “overweight” with a BMI between 25 and 29.9 kg/m^2^ and “obese” with a BMI greater than or equal to 30 kg/m^2^. The perception of the individual in relation to their level of physical activity during work and leisure time was reported. Subjects who reported remaining seated during the day and in their leisure time and did little or no exercise were classified as “inactive”; physical activity level “little” was used for those who reported remaining seated during the day and in their leisure time reported practice light to moderate intensity physical activity approximately twice a week; and physical activity level “regular” was used for those who reported practicing, at work or in their leisure time, moderate to intense physical activity three or more times a week. Regarding smoking habits, the categories were current smoker, ex-smoker and non-smoker.

Other independent variables included socio-demographic (age, gender and educational level), type of work at the airline company in four categories (administrative, blue collar, call center and air crew sector – involving pilots and flight attendants), and level of stress. The level of stress was assessed by individual self-perception on a Likert scale from zero to ten, in which zero represents “without stress” and ten is “the highest level of stress”. This variable was classified into “low stress” (score 0 to 3), “moderate stress” (4 to 6) and “high stress” (7–10).

The probability of developing coronary heart disease (CHD) over the next 10 years was calculated using the Framingham score [17]. For this calculation, points were assigned to the variables age, total cholesterol, systolic blood pressure and diabetes, stratified by sex. The total number of points was then converted into absolute risk for men and women. We considered as “higher risk” those who had at least 5% risk of developing CHD in the next 10 years.

### Data analysis

Descriptive analysis was used for the characteristics of the study population. To study how independent variables were interrelated, we used the chi-squared test and Spearman correlation. A multinomial logistic regression analysis was used to study associations of the dependent variable (with three categories comparing ‘1-9 days sick leave’ and ‘10 and more days sick leave’ with no sick leave) with socio-demographic, type of work and lifestyle-related factors. The odds ratio (OR) was estimated as measure of association with corresponding 95% confidence intervals (95% CI). In order to study the influence of educational level, type of work, stress, lifestyle factors, and risk for CHD on the association between gender and sick leave, these factors were added separately to the basic statistical model describing the association between gender and sick leave. All analyses were carried out with the STATA 12.0 statistical software package.

## Results

Table 1 describes the baseline characteristics, by gender, of the Brazilian workers at the airline company that was studied. The mean age of the employees was 32.2 years (SD = 8.4) and most of the subjects were male (59%). Compared to men, women in the study sample were younger, with lower educational level, concentrated in the ‘call center’ and ‘air crew’ types of work, and reported higher levels of stress in life. Both overweight and obesity were more frequent in men, as well as the risk for developing CHD. During the follow-up period, the occurrence of sickness absence was higher among women than men.

**Table 1:** Baseline characteristics among workers of an airline company (n = 2.150) by sex

	**Females**		**Males**		**Total**
	**n**	**%**	**n**	**%**	
**Individual characteristics**					
Age (years)					
Up to 29	497	56.4	449	35.4	946
30 - 39	300	34.0	520	41.0	820
40 - 49	72	8.2	213	16.8	285
50 or more	12	1.3	87	6.9	99
Educational level					
Even elementary school	802	91.3	788	62.1	1590
High school	8	0.9	160	12.6	168
College	71	8.1	321	25.3	392
Type of work					
Administrative jobs	298	33.8	279	22.0	577
Call center	212	24.1	98	7.7	310
Blue collar jobs	86	9.7	687	54.1	773
Air crew	285	32.3	205	16.1	490
Perceived stress in life					
Low	152	17.3	339	26.8	491
Moderate	326	37.1	485	38.3	811
High	400	45.6	441	34.9	841
**Lifestyle-related factors**					
Body mass index					
Normal weight (18.5-24.9)	632	71.7	480	37.8	1112
Overweight (25-29.9)	189	21.4	577	45.5	766
Obese (≥30)	60	6.8	212	16.7	272
Physical activity level					
Regular	130	14.7	281	22.1	411
Little	184	20.9	319	25.1	503
Inactive	567	64.4	669	52.7	1236
Smoking					
Non smoker	696	79.0	933	73.5	1629
Ex-smoker	100	11.3	166	13.1	266
Smoker	85	9.6	170	13.4	255
Absolute risk estimate for CHD*					
0%	661	92.2	488	43.9	1149
1-4%	45	6.2	391	35.2	436
> 5%	12	1.6	232	20.9	244
**Sick leave during 12 months**					
No sick leave	375	42.5	628	49.5	1003
1-9 days sick leave	328	37.1	467	36.8	795
10 or more days sick leave	178	20.4	174	13.7	352

*Absolute risk for coronary heart disease in the next 10 years based on the Framingham equation.

### Lifestyle factors

Strong differences were perceived on lifestyle-related factors between men and women. Men had a higher risk of developing CHD (OR = 26.14; CI = 15.45-47.24), were more likely to be overweight (OR = 4.02; CI = 3.28-4.92) or obese (OR = 4.65; CI = 3.41-6.34) and to be smokers (OR = 1.48; CI = 1.13-1.93). On the other hand, women were more likely to be less physically active in work and leisure time (OR = 1.82; CI = 1.43-2.30).

Regarding interrelation among the lifestyle-related factors, employees who were physically inactive more often had a high level of stress in life (OR = 2.14; CI = 1.60-2.88), were more likely to be obese (OR = 2.51; CI = 1.67-3.78) and to have a higher risk for CHD (OR = 1.70; CI = 1.13-2.58), compared to those with a regular level of physical activity. Ex-smokers were also more often obese than non-smokers (OR = 2.12; CI = 1.47-3.05). Obese workers were more likely to have increased risk for CHD (6.16; CI = 4.05-9.37) than non-obese.

### Sick leave

**Figure 1:**
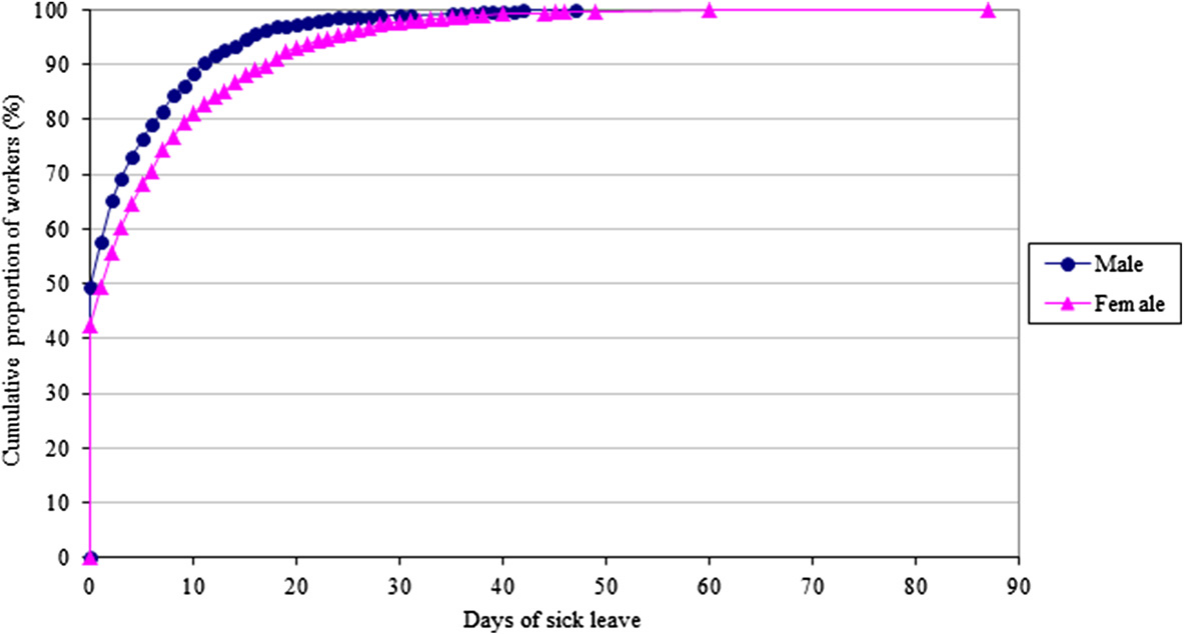
Sick leave distribution per cumulative proportion of workers.

During the 12 month follow-up period, 53.5% of the subjects had at least one sickness absence episode and among them, the average sick leave was 8.3 workdays. Considering the cumulative proportion of employees, we found that 54.2% of the employees of this study lost up to 1 day because of sick leave, 75.5% lost up to 6 days, and 90.6% lost up to 14 days. A small proportion (1.4% of the study population) lost more than 30 workdays as result of sickness. Figure 1 shows male and female distribution of days of sick leave by cumulative proportion of workers, stratified by sex. Cumulative sick leave between 8 and 20 days was much more common among women than men.

Table 2 shows, by univariate analysis, that women were more likely to have sick leave than men and this association was statistically significant for 10 or more days of sick leave. Older employees and those with higher educational levels had lower odds to have absenteeism. Regarding the type of work, employees with blue collar jobs were less likely to have 10 or more days of absenteeism. Those who worked as air crew were less likely to have 1–9 days of sick leave, but were more likely to have sick leave for 10 or more days. Workers with higher levels of stress were also more likely to have sick leave, and the odds was greater for them to have 10 or more days of absenteeism. By the univariate analysis, lifestyle-related factors were not associated with sick leave. Risk for CHD was associated with lower odds for 10 or more days of sick leave, but when this variable was adjusted for socio-demographic variables this association disappeared (OR = 1.27, CI = 0.67-2.42). When adjusted for socio-demographic variables, the odds for 10 or more days of sick leave increased and became significant both for smokers (OR = 1.51, CI = 1.05-2.17) and ex-smokers (OR = 1.45, CI = 1.01-2.10).

**Table 2:** Univariate odds ratios (OR) and 95% confidence intervals (CI) of socio-demographic characteristics and modifiable risk factors for sick leave among workers of an airline company (n = 2.150)

		**1-9 days sick leave (*n*** **= 811)**		**≥ 10 days sick leave (*n*** **= 366)**	
	**n**	**OR**	**95% CI**	**OR**	**95% CI**
**Individual characteristics**					
Gender					
Male	1274	1.00		1.00	
Female	927	1.17	0.97-1.42	1.70*	1.34-2.17
Age (years)					
Up to 29	982	1.00		1.00	
30 - 39	832	0.80*	0.64-0.96	0.71*	0.55-0.93
40 - 49	288	0.89	0.66-1.18	0.67*	0.45-1.00
50 or more	99	0.59*	0.37-0.96	0.39*	0.19-0.78
Educational level					
College	395	1.00		1.00	
High school	1638	1.49*	1.16-1.90	1.38*	1.01-1.90
Even elementary school	168	1.56*	1.06-2.30	0.64	0.34-1.20
Type of work					
Administrative	597	1.00		1.00	
Call center	324	0.92	0.68-1.24	1.03	0.70-1.52
Blue collar	779	0.87	0.69-1.10	0.62*	0.45-0.86
Air crew	501	0.74*	0.57-0.97	1.38*	1.00-1.90
Perceived stress in life					
Low	505	1.00		1.00	
Moderate	829	1.18	0.92-1.50	1.21	0.87-1.68
High	860	1.25	0.98-1.60	1.41*	1.02-1.94
**Lifestyle-related factors**					
Body mass index					
Normal weight (18.5-24.9)	1112	1.00		1.00	
Overweight (25-29.9)	766	0.88	0.72-1.08	0.61*	0.46-0.80
Obese (≥30)	272	0.81	0.61-1.09	0.74	0.51-1.08
Physical activity level					
Regular	416	1.00		1.00	
Little	506	0.86	0.64-1.14	1.08	0.74-1.57
Inactive	1279	1.07	0.84-1.36	1.21	0.88-1.68
Smoking					
Non smoker	1662	1.00		1.00	
Ex-smoker	268	1.24	0.92-1.64	1.30	0.91-1.87
Smoker	271	1.16	0.87-1.54	1.37	0.97-1.96
Absolute risk for estimate CHD**					
0%	1181	1.00		1.00	
1-4%	437	1.10	0.86-1.40	0.74	0.53-1.03
> 5%	245	0.74	0.54-1.01	0.64*	0.42-0.96

*p < 0,005.

**Absolute risk for coronary heart disease in the next 10 years based on the Framingham equation.

Women more often had sick leave, especially of 10 days or more (Table 2). Table 3 shows that after adjustment for type of work, the association between female gender and 10 or more days of sick leave decreased from OR = 1.70 to OR = 1.43 (16% change). Educational level also explained 7% of this association. Lifestyle-related factors did not influence the association between gender and sickness absence. After additional adjustment for all variables, the strength of the association between female gender and 10 or more days of sick leave was reduced another 39% and became non-significant.

**Table 3:** Effects of adjustment for educational level, stress, type of work, lifestyle factors and risk for CHD on the association between gender and sick leave

	**1-9 days sick leave**^**†**^		**10 or more days sick leave**^**†**^	
	**OR**^**‡**^	**95% CI**	**OR**^**‡**^	**95% CI**
Model 1: gender	1.17	0.97-1.42	1.70*	1.34-2.17
Model 2: model 1+ type of work	1.22	0.99-1.52	1.43*	1.09-1.87
Model 3: model 1 + educational level	1.12	0.92-1.35	1.57*	1.23-2.02
Model 4: model 1 + CHD^b^ risk	1.13	0.89-1.42	1.64*	1.21-2.22
Model 5: model 1 + stress	1.15	0.95-1.39	1.66*	1.30-2.12
Model 6: model 1 + lifestyle^a^	1.15	0.95-1.40	1.71*	1.34-2.20
Model 7: all variables	1.11	0.84-1.47	1.31	0.93-1.85

^**†**^Reference category: no sick leave.

^**‡**^Reference category: male.

^a^Lifestyle-related factors: overweight, obesity, physical activity, smoking.

^b^10-year absolute risk for coronary heart disease based on the Framingham equation.

*p < 0,05.

## Discussion

The current study aimed to analyze the relationship among lifestyle-related factors and sick leave and to examine whether gender differences in sickness absence can be explained by differences in type of work, educational level, and lifestyle factors. After adjustment for age, sex, and educational level, smokers had a significantly increased odds ratio for sick leave. Women were more likely to have more days of sick leave during the 12-month period. Strong differences in lifestyle were noted between men and women, but these differences did not influence the association of gender and sick leave. The association between gender and sick leave was explained, in part, by type of work and educational level.

With regard to lifestyle-related factors, the prevalence of obesity in our study (12.6%) was higher than that found in Brazilian industrial workers (7.9%) [18], but lower than the prevalence of obesity in a survey of adult populations in the city of São Paulo (15.5%) and Brazil (15.8%) [4]. In this study, 51.8% of workers reported that they were inactive at work and leisure time, corroborating previous reports of the lack of physical activity among Brazilian industrial workers [18]. The prevalence of smokers in the current study (12.3%) was also similar to that found in Brazilian industrial workers (13%), but lower than the prevalence of smokers among adults in Brazil (14.8%), and especially lower than the prevalence of smokers in São Paulo (22.5%) [4]. The risk of CHD was much higher among men compared to women. Studies have shown that differences in major cardiovascular risk factors, particularly in cholesterol level and smoking rate, explained a substantial part of the sex difference in CHD risk [17].

At the 1-year follow-up, 53.5% of the employees in our study lost at least one work day because of sickness. The prevalence of sickness absence was higher than in a Brazilian sample of automotive workers (39%) [19]. Different ways of measuring and categorizing sick leave make it difficult to engage in major comparisons between studies. In the current study, we found reduced absenteeism with increasing age. However, this association became non-significant after adjustment for educational level. It is interesting to observe that the associations between sickness absence and age and educational level were opposite to those commonly observed in studies from developed countries. Further research is needed to establish whether the reported associations are generalizable to other occupational populations in developing countries.

In our study, the only lifestyle-related factor associated with sick leave (10 or more days) was smoking, after adjustment for socio-demographic variables. This association is consistent with other studies [10,12,20] and with a recent meta-analysis that found a 33% increased risk of absenteeism in smokers compared with non-smokers [21]. The prevalence of smoking in people aged 18 years or older in Brazil has been considerably reduced in recent years, especially due to the ban on cigarette advertisements and the enacting of state laws that prohibit smoking in public or private indoor collective spaces. These developments have contributed to the observed prevalence of 12% smokers in our study population. In the current study, after adjustment for socio-demographic variables, ex-smokers were more likely to have 10 or more days of sick leave, compared with non-smokers. Other studies also found a greater prevalence of sick leave in ex-smokers compared with non-smokers [21-23], which suggests a need for organizations to not only encourage smoking cessation, but also to give support for former smokers.

Most studies on determinants of sick leave have been conducted among European and American workers. We found only two cross-sectional studies in Brazilian workers that investigated determinants of sick leave associated with lifestyle [19,24]. In the current study physical activity was not associated with sick leave. The association between physical activity and absenteeism is still not clear. While some studies show that workers with higher levels of physical activity lose fewer working days because of sickness [10,21], in other studies, this association is weak or inconsistent [12,19]. Several studies indicate an association between obesity and sick leave [10,24-26]. Our study did not find this association.

Similarly to what has been shown in several other studies [16,25,27], women in our study had higher sickness absence than men, and this difference was significant for 10 or more days of sick leave in the follow-up year. We analyzed the effects of adjustment for different variables on this association, and we found that the type of work accounted for 15% and educational level accounted for 7% of the association between female gender and 10 or more days of sick leave. Air crew workers, which consisted for 60% of women, had the highest risk for 10 or more days of sick leave. However, they had lower odds for 1–9 days of absenteeism. Air crew employees have some special characteristics that must be taken into account. For example, they are required to always be in a good physical condition, to be able to act in emergency situations. Indeed, the level of physical activity of air crew members in the current study was higher than in other types of work. However, the reason for the increased sick leave for this type of work may depend on several factors that were not examined.

Other researchers have shown that controlling by occupation partly explains gender differences in sick leave [15,16]. Laaksonen et al. [16] found that controlling for occupation explained approximately 33% of the gender difference in sickness absence episodes of more than two weeks in a sample of Finnish employees. In the current study, the educational level was lower in women. It is already known that workers with lower educational levels have a higher risk of sick leave [20,27]. Other factors, such as family situation (past reproductive history, small children at home, marital status or the like) were not measured in our study and may be relevant in explaining gender differences in sick leave [27].

The first limitation of this study is the non-representative sample. Only employees present in the company at the time of the assessment were considered, and it is possible that workers with poor lifestyle behaviors were absent from work on the assessment date. Second, measures of lifestyle-related factors were self-reported. Although the employees knew about the anonymity of their information, fear about being harmed in their jobs may have influenced their responses. Third, because the database of this study consisted of information that had already been collected, some information was limited, such as the stress and physical activity measurements. According to the literature recommendations [28], classifying the level of physical activity would require, in addition to information about intensity and weekly frequency, information about the duration of physical activity practice. Moreover, in the current study, work and leisure time activity were handled together, which may have had an effect on the results. Although the measurement were not optimal, these variables in our study had the expected associations and corroborated the prevalence in the Brazilian population. Thus, although limited, we believe that these variables are sufficiently valid to show an indication of the employees’ lifestyles. Fourth, sickness absence has a multifactorial nature. Although we adjusted the analyses for several factors, there may be confounders that were not taken into account.

The strengths of our study are its register-based data on sick leave and its prospective design with a relatively large sample of both female and male employees, which allowed us to draw more informed conclusions with respect to causality.

## Conclusion

The higher occurrence of sick leave among women than men was partly explained by type of work and educational level. Our results suggest that type of work, a stressful life, and smoking are important targets for health promotion in this study population, in order to reduce sickness absence and promoting workers’ health.

## Competing interests

The authors declare that they have no competing interests.

## Authors’ contributions

FMR participated in the design of the study, drafted the manuscript and performed the statistical analysis. RBL and PRM participated in the design of the study and checked the data. OCL and AMM participated in the design of the study and revised it critically. AB participated in performing the statistical analysis, drafted the manuscript and revised it critically. All authors participated in writing the manuscript and read and approved the final version.

## Pre-publication history

The pre-publication history for this paper can be accessed here:

http://www.biomedcentral.com/1471-2458/14/317/prepub
